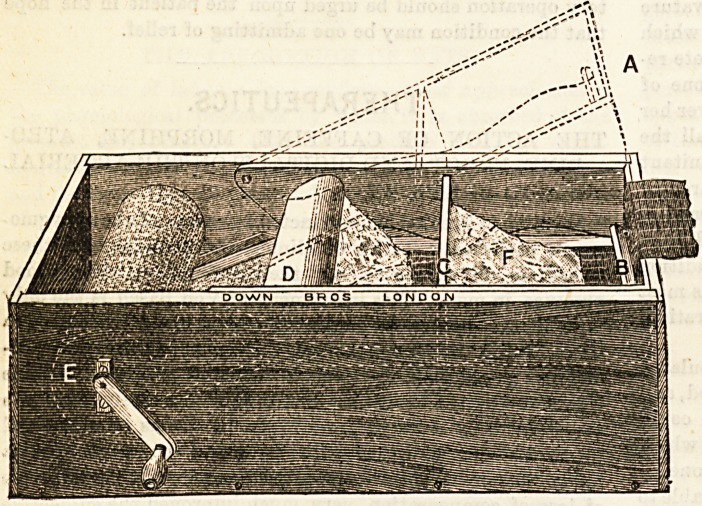# New Drugs, Appliances, and Things Medical

**Published:** 1891-06-20

**Authors:** 


### NEW DRUGS, APPLIANCES, AND THINGS MEDICAL.

[All preparations, appliances, novelties, etc., of which a notice is desired, should be sent for The Editor, to care of The Manager, 140, Strand, London, W.C.]

### A NEW PLASTER OF PARIS BANDAGE ROLLER.

Mr. E. Slaughter, B.A., M.D., of Brighton, writes as follows: The inconvenience of making plaster of paris bandages by hand induced me to try and make a machine, which, I think, will be found very useful, especially for hospital practice, and by surgeons practising in mining and manufacturing towns. The advantage attached to the machine may, I think, be summed up as being simplicity, moderate cost, rapidity in making the bandages, no loose parts to get lost, and any kind of bandage can be used; also it may be used as an ordinary bandage roller. The above illustration gives a very good idea of how the machine is worked. When a bandage is to be rolled raise the frame A (dotted line), and pass bandage over B and under C and D, close A and wind on to spinale E for a couple of turns. Place some plaster of paris, F, on the bandage as shown in the illustration, holding the bandage in the left hand and wind with right till finished. Messrs. Down Bros., of St. Thomas's Street, London, are the makers.

### A NEW BROWN BREAD.

Messrs. Paine and Co., St. Neot's, have sent us a sample of what is a new departure in the way of brown bread and biscuits. Brown bread, as usually met with in London, is nothing more than ordinary bread plus a certain quantity of bran, or it may sometimes contain in addition to these a proportion of the various siftings which result from the modern process of corn milling. Now, though London brown bread when fresh is a very pleasant article, it must be confessed that to many it, and a large proportion of whole-meal breads also, are indigestible. This is chiefly owing to the presence of the skins of the wheat clevels. Messrs. Paine have set to work in another way. During the process of milling as now done by the new methods recently brought into use, the "germ" of the seed is separated out from the various siftings and the flour which result from the grinding of the corn. The firm have returned the crushed germ to the flour, and from the bread and biscuits made from their combination the samples sent to us were made. Owing to accidental delay we did not receive them till they were six days old. The first thing we noticed was the remarkable freshness of the bread. An ordinary London brown loaf six days old would be quite dry and stale. The one under notice was quite eatable, in fact, reminding one of the condition of good home-made bread when of that age. The flavour is very pleasant, and both in the bread and biscuits a peculiar nutty character is distinguishable. Chemically we find both quite pure and of good food value. We consider Messrs. Paine have hit upon a distinctly serviceable and pleasant variety in the way of bread food stuffs, and can commend them thoroughly.

## Figures and Tables

**Figure f1:**